# Forecasting Non-Stationary Diarrhea, Acute Respiratory Infection, and Malaria Time-Series in Niono, Mali

**DOI:** 10.1371/journal.pone.0001181

**Published:** 2007-11-21

**Authors:** Daniel C. Medina, Sally E. Findley, Boubacar Guindo, Seydou Doumbia

**Affiliations:** 1 College of Physicians and Surgeons, Columbia University, New York, New York, United States of America; 2 Department of Population and Family Health, Mailman School of Public Health, Columbia University, New York, New York, United States of America; 3 Malaria Research and Training Center, Faculté de Médecine de Pharmacie et d'Odonto-Stomatologie (FMPOS), Université de Bamako, Bamako, Mali; London School of Hygiene & Tropical Medicine, United Kingdom

## Abstract

**Background:**

Much of the developing world, particularly sub-Saharan Africa, exhibits high levels of morbidity and mortality associated with diarrhea, acute respiratory infection, and malaria. With the increasing awareness that the aforementioned infectious diseases impose an enormous burden on developing countries, public health programs therein could benefit from parsimonious general-purpose forecasting methods to enhance infectious disease intervention. Unfortunately, these disease time-series often *i*) suffer from non-stationarity; *ii*) exhibit large inter-annual plus seasonal fluctuations; and, *iii*) require disease-specific tailoring of forecasting methods.

**Methodology/Principal Findings:**

In this longitudinal retrospective (01/1996–06/2004) investigation, diarrhea, acute respiratory infection of the lower tract, and malaria consultation time-series are fitted with a general-purpose econometric method, namely the multiplicative Holt-Winters, to produce contemporaneous *on-line* forecasts for the district of Niono, Mali. This method accommodates seasonal, as well as inter-annual, fluctuations and produces reasonably accurate median 2- and 3-month horizon forecasts for these non-stationary time-series, i.e., 92% of the 24 time-series forecasts generated (2 forecast horizons, 3 diseases, and 4 age categories = 24 time-series forecasts) have mean absolute percentage errors *circa* 25%.

**Conclusions/Significance:**

The multiplicative Holt-Winters forecasting method: *i*) performs well across diseases with dramatically distinct transmission modes and hence it is a strong general-purpose forecasting method candidate for non-stationary epidemiological time-series; *ii*) obliquely captures prior non-linear interactions between climate and the aforementioned disease dynamics thus, obviating the need for more complex disease-specific climate-based parametric forecasting methods in the district of Niono; furthermore, *iii*) readily decomposes time-series into seasonal components thereby potentially assisting with programming of public health interventions, as well as monitoring of disease dynamics modification. Therefore, these forecasts could improve infectious diseases management in the district of Niono, Mali, and elsewhere in the Sahel.

## Introduction

**Figure 1:**
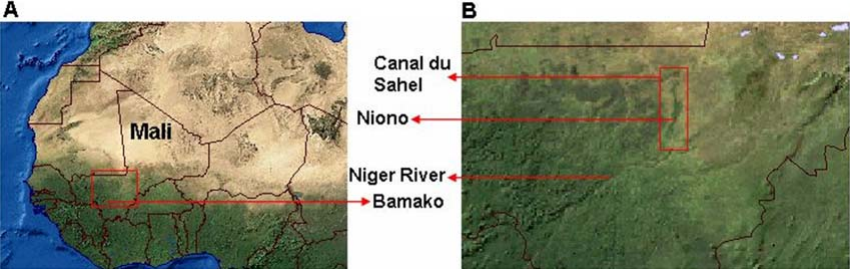
Map of West Africa. Panel A: The Sahara desert and the savannah occupy the northern and southern landscape, respectively, while the Sahel comprises the intermediate fringe zone. Mali lacks access to the ocean; its capital, Bamako, is indicated with a red pointer; and, the district of Niono is located inside the red demarcation, which is enlarged in panel B. Panel B: The black line on the top of this panel corresponds to the southeastern Mauritanian border; the depicted segment of the Niger River runs along the southwest-northeast direction; the district of Niono, which is located 330 km northwest of Bamako and 100 km north of the Niger River along the *Canal du Sahel*, is located within the red rectangle. This satellite image places the district of Niono in the Sahelian zone: poverty is extensive in the northern (semi-desert) and central (irrigated) regions; contrarily, poverty diminishes southward (savannah) where mixed crops prevail. *Image source*: adapted with permission from Globalis, http://globalis.gvu.unu.edu (08/2007).

Temperature and pluviometric fluctuations affect water resources, food production, and disease transmission [1]–[5]. In Africa, these fluctuations are coupled to *i*) the El Nino southern oscillator (ENSO), which impacts eastern and southern Africa the most; as well as, *ii*) the Atlantic dipole (AD) and the inter-tropical convergence zone (ITCZ) [6], which intimately govern climate thus potentially imparting famine and exacerbating disease transmission in the Sahel (**Figure 1**)—i.e., the sub-Saharan region that spans the entire east-west African axis, bordering the Sahara desert to the north and the Savanna to the south.

In much of the Sahel, climate-coupled diarrhea, acute respiratory infection of the lower tract (ARI), and malaria transmission continue to inflict most morbidity and mortality. Diarrhea transmission heightens during the rainy season presumably because of facilitated fecal contamination of water sources [7]–[10]. Diarrhea has multiple etiologies in the district of Niono. Viral diarrhea (e.g., *Rotavirus sp*.) is typical among infants; bacterial (e.g., *S. enterica*) and protozoan (e.g., *E. histolytica*) infection are more commonly diagnosed among older individuals. ARI incidence often culminates biannually during the dry season and again towards the end of the rainy season [1], [11]–[16]. ARI also has multiple etiologies in the district of Niono. Viral ARI (e.g., *Human respiratory syncytial virus*) is common among infants whereas, e.g., *H. influenza* and *S. pneumoniae* infection are more frequently diagnosed in older age categories. Last, malaria transmission behaves seasonally (or perennially) with a periodic intensification that follows the rainy season onset. *P. falciparum*, which is transmitted by the *Anopheles s.p.* vector, is the predominant pathogen (>99%) underlying malaria infection in Mali. Like diarrhea and ARI, the diagnosis of malaria is usually clinical in the district of Niono and much of the Sahel. Malaria affects those who have greater susceptibility to infection: *i*) children under 5 years of age who have not yet developed low-level immunity; *ii*) mal-nourished pregnant women; and *iii*) labor migrants from non-endemic zones who, like children under 5 years of age, lack low-level immunity [17]–[22]. Furthermore, pervasive malnutrition—which precedes harvesting and partially coincides with diarrhea, ARI, and malaria transmission zeniths—aggravates (and probably underlies) the somber epidemiological scenario crafted by the aforementioned diseases in the Sahel [7], [23], [24].

With the increasing awareness that infectious diseases impose an enormous burden on developing countries, public health programs therein could benefit from parsimonious general-purpose quantitative methods to enhance local intervention, i.e., diminish infectious disease-induced morbidity and mortality. Quantitative methods are particularly important for Sahelian countries—which rank among the poorest in the world—where the deployment of scarce resources is tantamount to a budgetary sacrifice. Consequently, disease risk assessment [25]–[30], epidemiologic forecasts [31]–[35], and early warning systems [36] are *de rigueur* before interventions can effectively mitigate the morbidity and mortality that infectious diseases inflict. In the context of forecasts, epidemiological time-series (TS) often *i*) exhibit inter-annual plus seasonal fluctuations; *ii*) require disease-specific tailoring of forecasting methods; and, *iii*) suffer from non-stationarity. [Stationary time-series (e.g., white-noise) have time-independent mean and covariance structures; conversely, non-stationary time-series (e.g., inventory) have time-dependent mean and covariance structures.]

**Figure 2:**
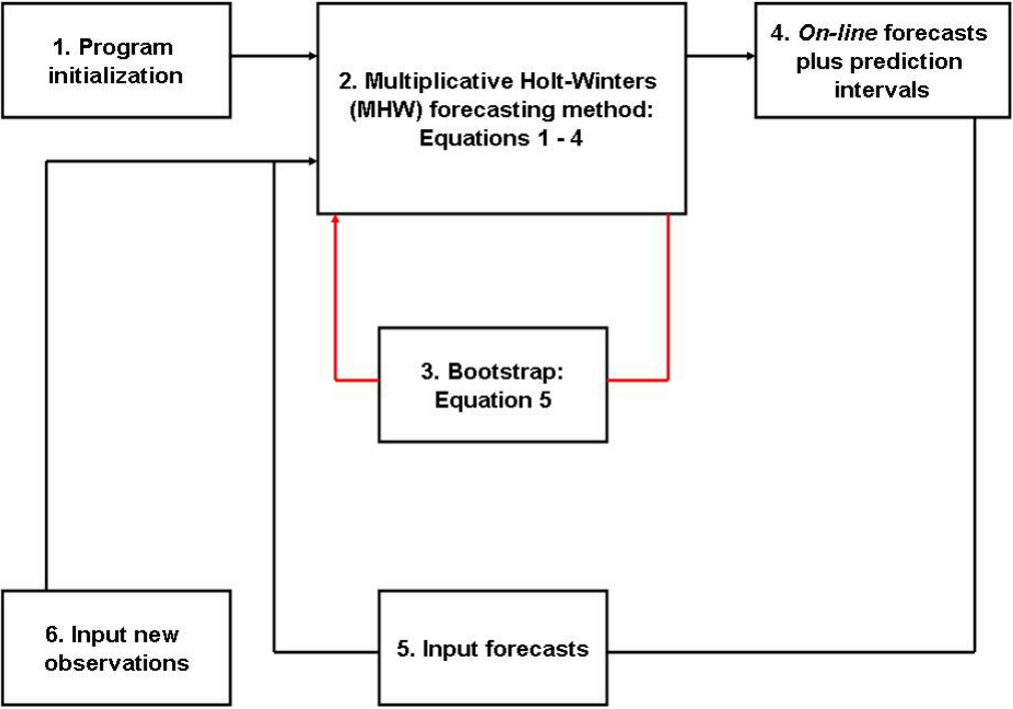
*On-line* forecast flow-chart. *On-line* forecasts imply that historical records are automatically and continuously supplied to the program, which revises forecasts. (1) Prior time-series observations and pseudo-parameter initialization are inputted into (2) the program that executes the multiplicative Holt-Winters (MHW) forecasting method, according to Equations 1–4 in the *Methods* section. (3) The program cycles (red arrow) through hundreds of residual bootstraps (Equation 5 in the *Methods* section) to produce (4) median forecasts and their 95% prediction interval bounds. Subsequently, (5) these forecasts plus (6) contemporaneous time-series observations are supplied to (2, 3) the MHW execution program, which revises (4) median forecasts and their prediction interval bounds. The automatic supply of contemporaneous time-series observations into (2–6) yields *on-line* forecasts.

Therefore, a general-purpose econometric method, namely the (seasonal) multiplicative Holt-Winters (MWH) [37], [38], is employed herein to forecast epidemiologically distinct non-stationary diarrhea, ARI, and malaria consultation rate TS for the district of Niono [39], [40], Mali (Fig. 1). The MHW method is extensively described in the *Methods* section and schematically depicted in **Figure 2**. This method ignores direct effects from climate (e.g., AD and ITCZ) on disease TS. Nevertheless, MHW-based historical TS analyses can obliquely capture non-linear prior interactions between climate and the aforementioned disease dynamics thus, potentially obviating the need for more complex disease-specific climate-based parametric forecasting methods (which are otherwise indispensable for highly-unstable transmission and or spatially-extended infectious disease models) in the district of Niono. This method could directly improve infectious disease management in this district with potential repercussions elsewhere in the Sahel where quantitative approaches may assist with predicting and containing infectious disease-induced morbidity and mortality.

## Results

### Consultation frequencies

Altogether, diarrhea, ARI, and malaria account for 59.2% of all consultations (333 990) during the investigational period (01/1996–06/2004) in the district of Niono [39], each perpetrating 7.5%, 13.2%, and 38.5% of total recorded morbidity, respectively (**Table 1**). A larger proportion of male than female individuals under 16 years of age are diagnosed with the aforementioned diseases; the converse is true for adults presumably because of differential risk-factor exposure. For comparison, Table 1 also displays the disease frequency distribution, based upon records from a single semester, for the district of Gao (last column) at approximately the same latitude but further east [41]. Though further investigations are necessary, the striking resemblance between these two record distributions further suggests epidemiological similarities across the Sahel. Consultation frequency distributions for the districts of Niono and Gao (Table 1) indicate that targeting diarrhea, ARI, and malaria is imperative to reduce morbidity (presumably mortality) in the district of Niono and possibly elsewhere in the Sahel. Of note, the reported malnutrition consultation frequency (0.9%) seems unrealistic when compared to the prevalence of stunted, under-weight, and wasted individuals in Mali, i.e., approximately 38%, 33%, and 11%, respectively [40].

**Table 1:** Disease consultation frequencies for the district of Niono.

Consultations	Frequency (%)									
	0–11 mo.		1–4 yr.		5–15 yr.		>15 yr.		Total (Niono)	Total (Gao)
	Male	Female	Male	Female	Male	Female	Male	Female		
**Malaria**	2.0	1.9	4.4	3.7	3.8	3.7	7.2	11.8	38.5	36.8
**ARI (lower)**	1.7	1.5	1.9	1.6	1.2	1.2	1.8	2.3	13.2	12.5
**Diarrhea**	1.1	1.1	1.3	1.0	0.5	0.5	0.9	1.1	7.5	4.8
**Traumatism, burn, injuries**	0.2	0.2	0.5	0.5	0.9	0.6	1.9	1.2	6.0	8.2
**ARI (upper)**	0.4	0.4	0.5	0.4	0.4	0.4	0.5	0.6	3.6	3.4
**Schistosomiasis**	<0.1	<0.1	0.1	0.1	0.7	0.4	0.7	0.4	2.5	0.8
**Problems during pregnancy**	-	-	-	-	-	<0.1	-	2.2	2.2	1.4
**Bubal/dental infection**	0.1	0.1	0.2	0.2	0.1	0.1	0.3	0.3	1.5	2.1
**Acute genito-urinary infection**	<0.1	<0.1	0.1	<0.1	0.1	0.1	0.5	0.7	1.5	1.5
**Genital ulcers**	<0.1	<0.1	<0.1	<0.1	<0.1	0.1	0.1	1.2	1.4	0.5
**Ocular infection**	0.1	0.1	0.1	0.1	0.1	0.1	0.3	0.3	1.4	2.2
**Malnutrition**	0.1	0.1	0.2	0.2	0.1	<0.1	<0.1	<0.1	0.9	0.3
**Problems during delivery/ post-partum**	-	-	-	-	-	<0.1	-	0.4	0.4	0.3
**Measles**	<0.1	<0.1	<0.1	0.1	0.1	0.1	<0.1	<0.1	0.3	<0.1
**Syphilis**	<0.1	<0.1	<0.1	<0.1	<0.1	<0.1	<0.1	0.1	0.2	3.7
**Trachoma**	<0.1	<0.1	<0.1	<0.1	<0.1	<0.1	<0.1	<0.1	0.1	0.1
**Meningitis**	<0.1	<0.1	<0.1	<0.1	<0.1	<0.1	<0.1	<0.1	0.1	<0.1
**Tuberculosis**	<0.1	<0.1	<0.1	<0.1	<0.1	<0.1	<0.1	<0.1	0.1	0.1
**Cholera**	<0.1	<0.1	<0.1	<0.1	<0.1	<0.1	<0.1	<0.1	0.1	0.5
**Tetanus**	<0.1	<0.1	<0.1	<0.1	<0.1	<0.1	<0.1	<0.1	<0.1	-
**Poliomyelitis**	<0.1	<0.1	<0.1	<0.1	<0.1	<0.1	<0.1	<0.1	<0.1	-
**Other**	1.1	1.0	1.3	1.1	1.3	1.3	4.6	6.6	18.3	20.9

Disease consultation frequencies for the district of Niono are expressed as percentages (%) from the total number of recorded consultations during the investigational period (01/1996–06/2004). Diarrhea, acute respiratory infection (ARI) of the lower tract, and malaria account for 59.2% of all consultations (333 990) that were recorded in the district of Niono [39] during this period. For comparison, the disease frequencies for the district of Gao (2005) are also displayed [41] in the last column. This table confirms that targeting diarrhea, ARI, and malaria is imperative to reduce morbidity, and presumably mortality, in the district of Niono, Mali.

### Diarrhea, ARI, and malaria consultation rates

Age category-specific median annual diarrhea, ARI, and malaria consultation rates, as well as corresponding inter-quartile range (IQR) values, for each of the 17 community health center (CSCOM) service areas within the district of Niono are reported in **Table 2**. Median annual consultation rates are expressed as the number of newly diagnosed cases *per* 1000 individuals in an age category during a year. Median annual consultation rates for younger age categories (Table 2) are disproportionately greater than their consultation frequencies (Table 1) because of age composition [40]. Generally, these rates diminish towards older age categories; hence, the systematic consultation rate age-dependence across CSCOM service areas emphasizes the need for age category-specific forecasts.

**Table 2:** Annual diarrhea, acute respiratory infection of the lower tract (ARI), and malaria consultation rates.

CSCOM	Median (IQR) annual consultation rate *per* 1000 individuals																							
	Diarrhea								ARI								Malaria							
	0–11 mo.		1–4 yr.		5–15 yr.		>15 yr.		0–11 mo.		1–4 yr.		5–15 yr.		>15 yr.		0–11 mo.		1–4 yr.		5–15 yr.		>15 yr.	
**Boh**	48	(21)	27	(9)	4	(3)	16	(9)	108	(10)	45	(20)	15	(6)	25	(7)	157	(139)	64	(33)	28	(14)	58	(21)
**Bolibana**	65	(13)	30	(13)	9	(4)	5	(2)	168	(48)	75	(14)	38	(12)	15	(5)	134	(80)	85	(18)	49	(13)	36	(15)
**Cocody**	135	(69)	44	(25)	8	(8)	10	(4)	193	(162)	67	(77)	26	(27)	15	(13)	172	(87)	150	(55)	78	(31)	99	(56)
**Debougou**	193	(80)	41	(20)	8	(3)	6	(4)	207	(25)	52	(18)	23	(5)	15	(3)	145	(38)	74	(21)	53	(14)	52	(19)
**Diabaly**	85	(35)	25	(16)	3	(3)	9	(6)	125	(33)	36	(21)	12	(8)	20	(10)	102	(27)	88	(35)	57	(15)	75	(11)
**Diakiwere**	67	(27)	31	(12)	7	(7)	7	(5)	102	(40)	53	(20)	15	(10)	12	(4)	203	(73)	147	(52)	86	(43)	71	(16)
**Dogofry**	169	(54)	27	(7)	3	(2)	6	(4)	237	(71)	38	(38)	12	(3)	15	(10)	272	(196)	143	(51)	57	(18)	113	(45)
**Fassoun**	128	(NA)	50	(NA)	9	(NA)	12	(NA)	230	(NA)	60	(NA)	14	(NA)	15	(NA)	206	(NA)	134	(NA)	60	(NA)	69	(NA)
**Kourouma**	58	(21)	17	(11)	1	(1)	1	(1)	207	(45)	62	(17)	19	(9)	24	(8)	142	(35)	102	(17)	42	(8)	68	(12)
**Molodo**	187	(18)	52	(7)	13	(2)	16	(3)	372	(187)	121	(24)	48	(12)	54	(10)	412	(194)	181	(23)	87	(30)	179	(34)
**Nampala**	12	(12)	12	(10)	1	(1)	4	(4)	25	(28)	11	(12)	4	(3)	8	(5)	53	(31)	32	(7)	15	(7)	29	(6)
**Nara**	35	(NA)	4	(NA)	0	(NA)	0	(NA)	105	(NA)	16	(NA)	3	(NA)	3	(NA)	116	(NA)	29	(NA)	9	(NA)	26	(NA)
**Pogo**	58	(22)	26	(4)	2	(2)	9	(1)	132	(32)	57	(31)	17	(8)	22	(5)	192	(87)	112	(39)	30	(17)	51	(10)
**Siribala**	120	(54)	32	(6)	4	(1)	6	(1)	214	(50)	59	(18)	17	(6)	15	(4)	148	(79)	96	(47)	44	(22)	58	(4)
**Sokolo**	152	(42)	51	(26)	16	(11)	15	(7)	397	(130)	104	(19)	57	(23)	35	(19)	282	(143)	185	(44)	102	(33)	102	(40)
**Werekela**	153	(97)	32	(38)	11	(5)	8	(1)	223	(118)	58	(14)	29	(6)	16	(7)	532	(223)	194	(85)	78	(35)	77	(18)
**Niono**	166	(NA)	32	(NA)	8	(NA)	7	(NA)	408	(NA)	97	(NA)	36	(NA)	21	(NA)	203	(NA)	119	(NA)	73	(NA)	58	(NA)

Median annual consultation rates (and inter-quartile ranges, i.e., IQR) are tabulated for each community health center (CSCOM) service area and age category: 0–11 months, 1–4 years, 5–15 years, and >15 years. Consultation rates are calculated, and expressed, as the number of newly diagnosed cases *per* 1000 individuals in an age category during a year (decimal places omitted). IQR values could not be estimated (NA) for Fassoum, Nara, and Niono CSCOM service areas because of limited record availability (*Methods*). Generally, median annual consultation rates diminish towards older age categories, demonstrating the need for age category-specific forecasts. Of note, the Niono CSCOM service area, which includes the district center and immediate periphery, is one of the 17 CSCOM service areas within the district of Niono, Mali.

A non-parametric ‘analog’ of the coefficient of variance, i.e., the ratio of IQR-to-median values, robustly measures annual consultation rate dispersion for each CSCOM service area. Notwithstanding the large median annual consultation rate variability across CSCOM service areas, CSCOM-specific ratios of IQR-to-median values are also large (not shown). Consequently, spatially-temporal forecasts for the district of Niono seem unnecessary in this initial developmental phase.

### Time-series forecasts

**Figure 3:**
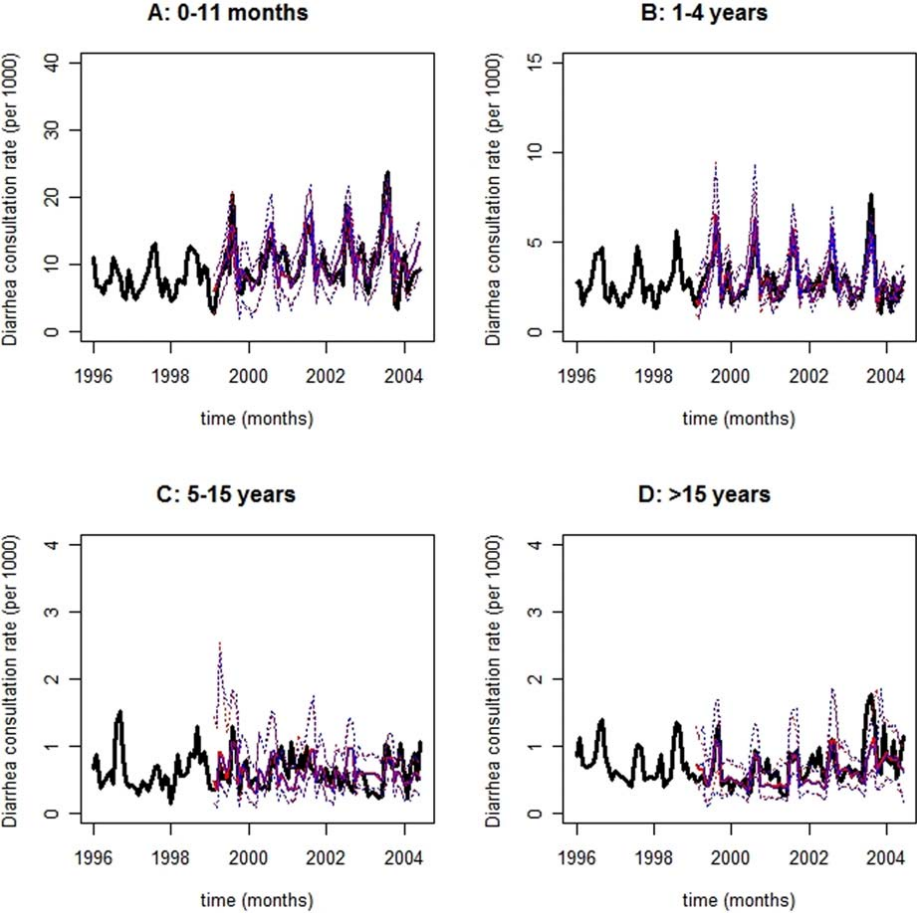
Forecasts for diarrhea consultation rate time-series. Observed diarrhea consultation rate time-series are depicted as black lines while red and blue traces correspond to contemporaneous 2- and 3-month horizon forecasts, respectively; their 95% prediction interval bounds are symbolized by dots of the same colors. Forecasts and prediction interval bounds are calculated with a bootstrap-coupled seasonal multiplicative Holt-Winters method (*Methods*). In each panel, the abscissa spans 102 months (01/1996–06/2004) while the ordinate represents the number of newly diagnosed cases *per* 1000 age category-specific individuals. Panel A: 0–11 months; Panel B: 1–4 years; Panel C: 5–15 years; and, Panel D: >15 years. Forecasting accuracy is greater for younger age categories where seasonality dominates and mortality is highest. Of note, age category-specific 2- and 3-month horizon diarrhea consultation rate forecasts roughly overlap because of slowly shifting level and negligible trend time-series components. Consequently, 2- and 3-month horizon 95% prediction interval bounds also overlap.

**Figure 4 pone-0001181-g004:**
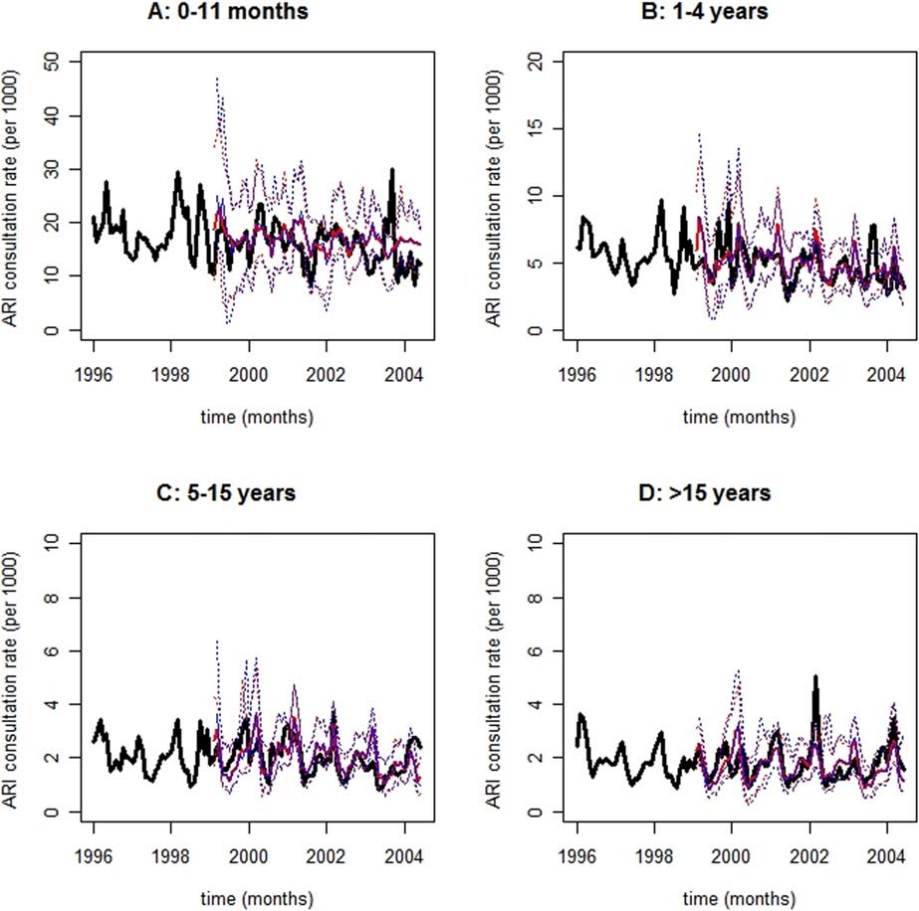
Forecasts for acute respiratory infection (ARI) consultation rate time-series. Observed ARI consultation rate time-series are depicted as black lines while red and blue traces correspond to contemporaneous 2- and 3-month horizon forecasts, respectively; their prediction interval bounds are symbolized by dots of the same colors. Forecasts and prediction interval bounds are calculated with a bootstrap-coupled seasonal multiplicative Holt-Winters method (*Methods*). In each panel, the abscissa spans 102 months (01/1996–06/2004) while the ordinate represents the number of newly diagnosed cases *per* 1000 age category-specific individuals. Panel A: 0–11 months; Panel B: 1–4 years; Panel C: 5–15 years; and, Panel D: >15 years. Forecasts deteriorate slightly towards older age categories owing to seasonality attenuation. Of note, age category-specific 2- and 3-month horizon ARI consultation rate forecasts roughly overlap because of slowly shifting level and negligible trend time-series components. Consequently, 2- and 3-month horizon 95% prediction interval bounds also overlap.

**Figure 5:**
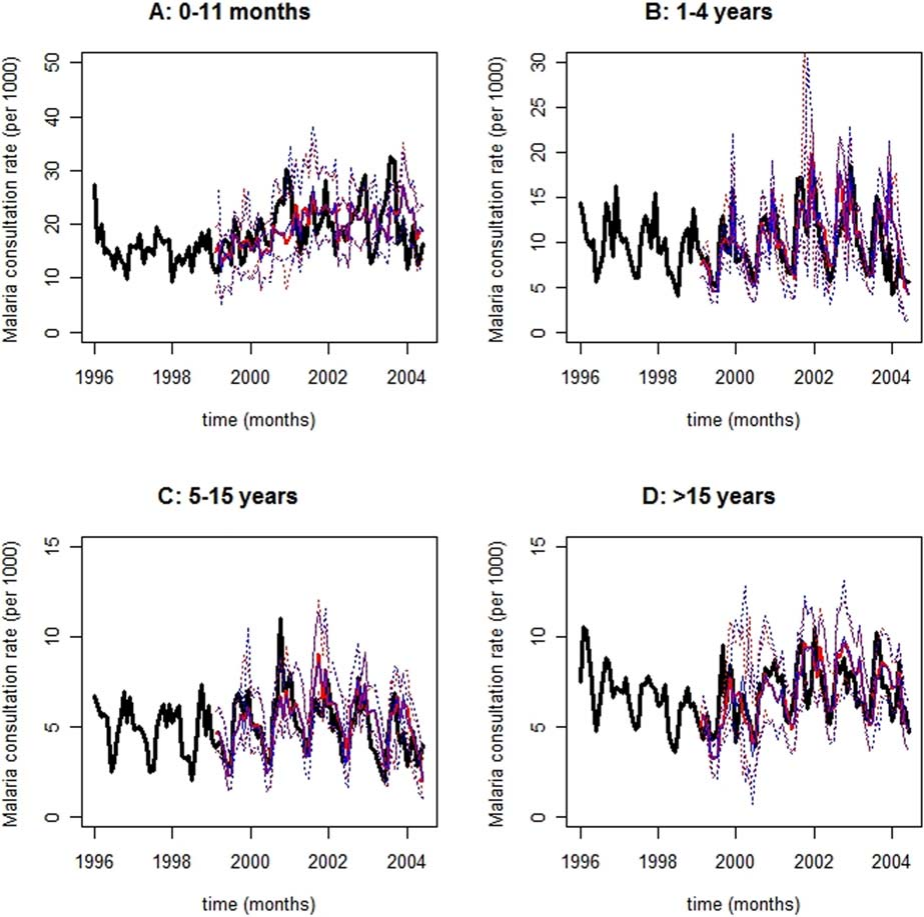
Forecasts for malaria consultation rate time-series. Observed malaria consultation rate time-series are depicted as black lines while red and blue traces correspond to contemporaneous 2- and 3-month horizon forecasts, respectively; their prediction interval bounds are symbolized by dots of the same colors. Forecasts and prediction interval bounds are calculated with a bootstrap-coupled seasonal multiplicative Holt-Winters method (*Methods*). The abscissa spans 102 months (01/1996–06/2004) while the ordinate represents the number of newly diagnosed cases *per* 1000 age category-specific individuals. Panel A: 0–11 months; Panel B: 1–4 years; Panel C: 5–15 years; and, Panel D: >15 years. Forecasts ameliorate towards older age categories owing to seasonality accentuation. Visual inspection suggests a resemblance between ARI (Fig. 4) and malaria time-series, as well as forecasts, for the youngest age category (Panel A: 0–11 months), which might reflect misdiagnosis and or co-morbidity between these two diseases. Clinical diagnosis among infants suffers from two major limitations. Infants are *i*) immunologically complex and *ii*) unable to effectively communicate symptoms. Of note, age category-specific 2- and 3-month horizon malaria consultation rate forecasts roughly overlap because of slowly shifting level and negligible trend time-series components. Consequently, 2- and 3-month horizon 95% prediction interval bounds also overlap.

The MHW forecasting method has been described in the *Methods* section and schematically depicted in Fig. 2. MHW Equation 1 produces point forecasts, which are recursively revised with Equations 2–4, whilst their 95% prediction interval bounds (PI) are estimated with Equation 5. Eqs. 1–5 also decompose TS into level, trend (rate of change), seasonal, and approximately serially uncorrelated residual TS components, i.e., {*l_t_*}, {*r_t_*}, {*s_m_*_|*t*_}, and {*z_t_*}, respectively. MHW pseudo-parameters *α*, *β*, and *γ* (Eqs. 2–4) control exponential smoothing of corresponding *l_t_*, *r_t_*, and *s_m_*_|*t*_ TS components. In **Figures 3** **–** [Fig pone-0001181-g004] **5**, observed age category-specific amalgamated consultation rate TS for diarrhea (Fig. 3), ARI (Fig. 4), and malaria (Fig. 5) are symbolized by black lines while red and blue traces correspond to contemporaneous *on-line* (fig. 2) median 2- and 3-month horizon (*h*) MHW forecasts, respectively; their 95% PI values are depicted in dots of the same colors. Abscissa TS projections span 102 months (01/1996–06/2004) while ordinate values represent the number of newly diagnosed cases *per* 1000 age category-specific individuals. TS ordinate scales diminish with increasing age across panels A (0–11 months), B (1–4 years), C (5–15 years), and D (>15 years), reflecting the age-dependent consultation rate distribution elucidated in Table 2.

Malaria (Fig. 5) and diarrhea (Fig. 3) consultations exhibit the largest and smallest inter-annual fluctuations, respectively, as intuited from their consultation rate TS (black lines) and implied by their decomposed {*l_t_*} plus {*r_t_*} TS components (not shown). Consultation rate TS evolution (black lines) also reveals dominant seasonal oscillations in the transmission of diarrhea, ARI, and malaria (Figs. 3–5). The strength of MHW pseudo-parameters mostly follow *γ*≫*α*≥*β*, which obtain for highly seasonal TS with negligible *r_t_* components (**Table 3**). Disease- and age category-specific 2- and 3-month horizon forecasts roughly overlap because decomposed {*l_t_*} shifts slowly and {*r_t_*} approaches nil. Consequently, 2- and 3-month horizon 95% PI values also overlap. Generally, TS forecasts (Figs. 3–5) are accurate and precise, varying degrees of seasonal and inter-annual TS fluctuations notwithstanding.

**Table 3:** Multiplicative Holt-Winters method: pseudo-parameter and mean absolute percentage error (MAPE) values.

Diseases	Age	*α*		*β*		*γ*		MAPE (%)			
								*h* = 2		h = 3	
								MHW	SA_3_	MHW	SA3
**Diarrhea**	**0–11 mo.**	0.01	(0.03)	<0.01	(0.16)	0.35	(0.14)	26.6	30.3	25.3	30.1
	**1–4 yr.**	0.01	(0.02)	<0.01	(0.04)	0.32	(0.16)	23.2	30.3	22.8	30.2
	**5–15 yr.**	<0.01	(0.02)	0.02	(0.21)	0.32	(0.17)	42.4	43.3	43.5	44.1
	**>15 yr.**	0.11	(0.19)	0.03	(0.15)	0.23	(0.17)	26.2	32.9	27.0	33.8
**ARI**	**0–11 mo.**	0.21	(0.32)	<0.01	(<0.01)	0.24	(0.21)	25.2	25.7	25.0	26.8
	**1–4 yr.**	0.03	(0.07)	<0.01	(0.02)	0.31	(0.19)	22.5	28.2	22.9	30.0
	**5–15 yr.**	0.07	(0.09)	<0.01	(<0.01)	0.30	(0.15)	25.9	25.3	26.3	26.7
	**>15 yr.**	0.23	(0.30)	<0.01	(<0.01)	0.30	(0.20)	24.1	27.1	25.0	30.0
**Malaria**	**0–11 mo.**	0.08	(0.09)	<0.01	(<0.01)	0.27	(0.14)	22.9	23.2	22.4	25.9
	**1–4 yr.**	0.18	(0.22)	<0.01	(0.22)	0.24	(0.13)	23.1	28.4	22.9	30.5
	**5–15 yr.**	0.09	(0.09)	<0.01	(0.06)	0.30	(0.16)	22.7	20.9	22.4	23.2
	**>15 yr.**	0.13	(0.08)	0.01	(0.12)	0.26	(0.12)	17.8	17.5	18.1	18.1

Median (and inter-quartile range) pseudo-parameter *α*, *β*, and *γ* values—which smooth control level, trend, and seasonal time-series components, respectively—reflect fitting of *B* = 500 bootstrap-generated full-length pseudo-time-series with the seasonal multiplicative Holt-Winters method. The greater the pseudo-parameter value, the shorter the smoothing memory, i.e., information from the recent-past have more pronounced effects on estimates than those from the distant-past. Generally, the strength of pseudo-parameters follows *γ*≫*α*≥*β*, which is expected for time-series with highly seasonal and negligible trend components. Furthermore, large mean absolute percentage error (MAPE) values between observed monthly consultation rates and their median forecasts imply low accuracy and *vice-versa*. Thus, 92% of the 24 time-series (TS) forecasts generated here (2 forecast horizons, 3 diseases, and 4 age categories = 24 TS forecasts) are reasonably accurate, i.e., their MAPE values are *circa* 25%. MAPE values from a seasonal adjustment (SA_3_) forecasting method [32] are also listed for benchmark comparison. The MHW performance is equal or superior to that of the SA_3 _forecasting benchmark in 87.5% of the 24 TS forecasts generated here, as implied by equal or smaller MAPE values.

Forecasts for ARI (Fig. 4) and malaria (Fig. 5) tend to perform better than those for diarrhea (Fig. 3) consultation rate TS (Table 3). Forecasts for ARI (Fig. 4) and malaria (Fig. 5) consultation rate TS deteriorate and ameliorate (though only faintly), respectively, towards older age categories. Furthermore, forecasts for diarrhea consultation rate TS (Fig. 3) are reasonably accurate for panels A (0–11 months), B (1–4 years), and D (>15 years) where seasonality dominates. Beyond visual inspection of Figs. 3–5, forecasting accuracy is assessed with mean absolute percentage error (MAPE) values between observed monthly consultation rates and their median forecasts (Table 3). These revolve *circa* 25% regardless of disease, age category, or forecasting horizon. The exceptions are: *i*) the low forecasting accuracy for the diarrhea consultation rate TS that appears in panel C (5–15 yr.) of Fig. 3, i.e., 42.4% and 43.5% for *h* = 2 and *h* = 3, respectively; and, *ii*) the high forecasting accuracy for the malaria consultation rate TS that appears in panel D (>15 yr.) of Fig. 5, i.e., 17.8% and 18.1% for *h* = 2 and *h* = 3, respectively. Notice that 92% of the 24 TS forecasts generated (2 forecast horizons, 3 diseases, and 4 age categories = 24 TS forecasts) are reasonably accurate, i.e., their MAPE values are *circa* 25% (Table 3). Additionally, narrow forecasting PI values reflect a high prediction precision. In all cases, *a priori* Box-Cox TS transformations fail to improve forecasting accuracy and precision.

Finally, Table 3 also compares the performance of the MHW method against that of a seasonal adjustment (SA_3_) forecasting benchmark, which was recommended by Abeku *et al.* [32], and which has been briefly described in the *Methods* section. The MHW performance is equal or superior to that of the SA_3 _forecasting benchmark in 87.5% of the 24 TS forecasts generated here, as implied by equal or smaller MAPE values (Table 3). Statistically, the location of SA_3_-to-MHW MAPE value ratios (*c.* 1.13) is greater than unity (Wilcoxon test: *p-value*<0.001) regardless of disease, age category, or forecasting horizon. Furthermore, the average intra-method MAPE value ratio between consecutive horizons estimates the overall forecasting horizon crude deterioration rate (CDR). SA_3 _and MHW CDR values are approximately 1.053 and 1.002 *per* horizon, respectively. Consequently, SA_3 _predictions deteriorate faster than MHW forecasts as the horizon *h* increases.

## Discussion

The descriptive statistics (Tables 1 & 2) presented in the *Results* section demonstrate the necessity for age category-specific monthly diarrhea, ARI, and malaria consultation rate TS forecasts in the district of Niono, Mali (Fig. 1). The majority (92%) of these MHW-based disease TS forecasts are reasonably accurate (i.e., their MAPE values are *circa* 25%) regardless of forecasting horizon (*h* = 2 & *h* = 3), disease, or age category (Figs. 3–5 & Table 3). The MHW performance is equal or superior to that of the SA_3_ forecasting benchmark [32] in 87.5% of the 24 TS forecasts generated here, as implied by equal or smaller MAPE values (Table 3). Notice that forecast adjustment with deviations between recent predictions and their observed TS values is a common feature to both MHW and SA_3_ methods. However, the MHW method exponentially weighs the full TS length unlike the SA_3_ method, which arbitrarily truncates information.

Notwithstanding the paucity of published MHW-based infectious disease TS forecasting investigations, these results (Figs. 3–5 & Table 3) suggest that the exceptional performance of the MHW method in econometrics and beyond since the 1950s [37], [38], [42]–[46] may be capitalized by the public health sector. The generality, reasonable performance, and operational simplicity of the MHW forecasting method may appeal to those working towards infectious disease hazard mitigation. Particularly, this investigation could assist authorities with early-warning the public and mitigation capacities before infectious disease calamities in the district of Niono and possibly elsewhere in the Sahel.

The search for general-purpose disease TS forecasting methods parallels the econometric quest for robust approaches to predict, e.g., inventory. Thus, this public health pursuit is herein addressed with the MHW forecasting method. This method *i*) adapts to underlying alterations in disease TS dynamics and *ii*) predicts disease TS with distinct epidemiological signatures (diarrhea: oral-fecal transmission; ARI: conjunctival, nasal, or oral transmission; malaria: vector transmission) as confirmed by Figs. 3–5. The MHW method adaptability emanates from the *on-line* training that exponentially discounts prior information, i.e., information from the recent-past is more relevant to forecasts than those from the distant-past. Its versatility reflects ‘density-estimation’ of {*l_t_*}, {*r_t_*}, and {*s_m_*_|*t*_} (*Methods*). Owing to both adaptability and versatility, the MHW method tends to accommodate intervention-induced perturbations (e.g., prophylaxis and medical treatment) that inherently plague longitudinal retrospective disease TS investigations. It handles TS trend, seasonal and inter-annual fluctuations, as well as long-term seasonality attenuation (or accentuation). The latter remark remains particularly pertinent to disease TS forecasts for the youngest, and most vulnerable, age categories (Figs. 3–5 & Table 3). Furthermore, the aforementioned MHW properties are fundamental to accommodate climate-dependent seasonal and inter-annual disease TS fluctuations. Whether the MHW method may respond to climate-unrelated between-year disease transmission signals remains *terra incognita*.

In the Sahel, small ITCZ position shifts can impart large pluviometric, and consequent disease TS, fluctuations. Malaria consultation rate TS (Fig. 5) exhibit greater ITCZ-mediated inter-annual fluctuations than diarrhea (Fig. 3) or ARI (Fig. 4) consultation rate TS presumably because malaria transmission is vector-amplified thus, more tightly coupled to environment and climate. For instance, a slight pluviometric increase may expand *Anopheles sp*. breeding sites (and consequent malaria transmission) considerably whereas a disproportionately larger precipitation could delay (or suppress) malaria transmission owing to partial (or complete) breeding site obliteration. On the other hand, a slight pluviometric increase insignificantly enhances oral-fecal exposure (and consequent diarrhea infection) whereas a larger precipitation may appreciably facilitate diarrhea transmission. Despite distinct climate-mediated disease TS fluctuations in the district of Niono, the MHW method forecasts non-stationary diarrhea, ARI, and malaria consultation rate TS (Figs. 3–5) with reasonable accuracy (Table 3) and without disease-specific tailoring of the forecasting method.

Typically, seasonal and inter-annual disease consultation rate TS fluctuations mirror prior climate and disease dynamics coupling. Methods that ‘accommodate’ prior climate and disease dynamics coupling tend to reflect it on post-sample forecasts. The MHW method captures these interactions (under partially- or fully-stable transmission), decomposing highly non-linear TS features (e.g., seasonal components) and hence, ascribing a non-linear ‘flavor’ to disease TS forecasts.

This method also de-correlates {*l_t_*}, {*r_t_*}, {*s_m|t_*}, and {*z_t_*} features, which may be employed to investigate long-term disease TS trend and seasonality. Seasonal factors, i.e., {*s_m|t_*}, do not differ dramatically in subsequent years because intermediate-frequency (seasonal) climate patterns tend to shift only slowly, if at all, in the district of Niono. Furthermore, seasonal pattern estimates are revised *on-line* and hence, mitigating possible discrepancies between observed seasonality and its post-sample projections. The stability of seasonal TS components is not trivial. While forecasts allow the public health sector to timely emerge with tailored disease interventions, extracted seasonal patterns may support development of robust programmatic public health interventions and personnel training. Furthermore, long-term seasonality analysis could assist with monitoring of: *i*) climate-induced disease dynamics alterations; and *ii*) environmental modification strategies to obviate or attenuate, e.g., malaria transmission [47]. A comprehensive seasonal {*s_m_*_|*t*_} analysis to support programmatic public health intervention, personnel training, and disease dynamics modification in the district of Niono remains in progress (Findley SE, Doumbia S, Medina DC, Guindo B, Toure MB, Sogoba N, Dembele M, & Konate D. Season-smart: how knowledge of disease seasonality and climate variability can reduce childhood illnesses in Mali. Proceedings of IUSSP-XXV International Population Conference. Tours, France, 2005).

Additionally, residual {*z_t_*} analysis may assist with adjusting forecasts amid sudden TS fluctuations, to which essentially all univariate TS forecasting methods are vulnerable. At best, univariate methods, such as the MHW, react after initial TS perturbations evolve. This limitation may be partially circumvented with educated pseudo-parameter adjustments in MHW Eqs. 2–4 as well as ‘expert’ re-initializations, presumably reflecting recent TS fluctuations. Moreover, educated adjustment may partially anticipate TS alterations when sufficient *a priori* knowledge from exogenous factors is available. [Regardless of adjustment adequacy, stable forecasts resume only upon TS instability termination.] A promising approach to decide whether such educated adjustments are imperative relies on inspection of serially uncorrelated ‘outlying’ {*z_t_*} TS values [42].

Computationally, the recursive MHW method may be easily optimized and operated by non-statisticians in the public health sector. The MHW and other exponential smoothing forecasting methods are available as software procedures (e.g., SPSS® & EViews®), pre-written functions for programming environments (e.g., S-plus® & the freely-available R language and environment for statistical computing), and codes in classical programming languages (e.g., FORTRAN & C) among myriad options. Exponential smoothing TS forecasts easily obtain with the user-friendly SPSS® and EViews®. Nevertheless, forecasting quality inevitably depends on the optimization algorithm that each application employs. Real-world application of most forecasting methods, including exponential-smoothing, require varying degrees of programming for *on-line* and bootstrapping implementation. Furthermore, the operational simplicity of the MHW method contrasts the sophistication and complexity of models such as the seasonal autoregressive integrated moving-average (SARIMA) as well as lagged weather- and or climate-based regression models. [Of note, climate is a statistic (e.g., mean monthly precipitation) whereas weather is a chaotic event (e.g., daily precipitation).]

The statistically more sophisticated, and also widely available, SARIMA model fits disease TS but, not only has it reportedly failed to consistently produce robust post-sample forecasts for malaria TS in Abeku *et al.* [32] but it may also require extensive user expertise for optimum performance. SARIMA models require users to iteratively specify SARIMA (*p*, *d*, *q*)(*P*, *D*, *Q*) components, enforce post differencing stationarity, and test for residual normality until an acceptable model specification is achieved. The orders *p*, *d*, *q*, *P*, *D*, and *Q* symbolize the number of auto-regressive lags, differences, moving-average lags, seasonal auto-regressive lags, seasonal differences, and seasonal moving-average lags, respectively. The SARIMA approach not only lacks a systematic procedure to uniquely specify SARIMA (*p*, *d*, *q*)(*P*, *D*, *Q*) components but it also requires a minimum of 50 to 100 observations for optimization. Conversely, the MHW forecasting method implemented herein requires a minimum of 36 TS observations plus pseudo-parameter initialization (e.g., *α* = *β* = *γ* = 0.3) for optimization. This advantage is vital when available monthly records span 10 years or less, such as the 102 composite monthly disease TS observations that are reported and analyzed in this manuscript. Unlike SARIMA, however, the MHW method is not a statistical model since it lacks an error structure. Hence, PI estimation is herein accomplished *via* bootstrapping rather than analytically. More sophisticated state-space exponential smoothing alternatives [43]–[46] may surpass the performance of MHW-based TS forecasts and improve 95% PI value estimation.

Lagged weather- and or climate-based models are particularly powerful whenever disease transmission is highly-unstable and epidemics are suddenly-triggered. A weather-based Poisson regression (4^th^-degree polynomial distributed lag) model has successfully forecasted malaria TS in highly unstable regions of Ethiopia [35]. However, lagged weather- and or climate-based models not only require extensive programming and user expertise to reasonably specify the number of lags for each model component but they also suffer from multicollinearity, problematic optimization, and lengthy TS requirements. Furthermore, lagged models, unlike the MHW method, must be tailored to each disease. While some form of lagged weather- and or climate-based model may be indispensable [35], the simpler MHW alternative may forecast fully- or partially-stable disease TS, e.g., *meso*-endemic malaria transmission in the district of Niono. Only under the most unstable transmission conditions should climate and or weather covariates be invoked to forecast disease TS at specific locations [32]–[35]. Weather events must be measured because predicting its chaotic nature with several weeks in advance is usually impossible. Predicting climate is not trivial and such predictions are typically too global to substantially add local forecasting accuracy [32]. Otherwise, climate-based models have paramount importance in infectious disease investigations to: forecast long horizons [48], elucidate complex disease transmission behavior [34], [48], and model infectious disease transmission in the spatial dimension [36].

Forecasting approaches perform reasonably well whenever disease transmission comprises relatively large event-probabilities during (long) investigational periods. Like other forecasting approaches, the MHW method surrenders when disease transmission depends on rare stochastic events (in highly-structured populations), each associated with minute (albeit finite) probabilities, governing transient disease dynamics [49]. These highly-stochastic structured disease dynamics feature sudden epidemic resurgence and ample epidemic volume variability that are not easily investigated with univariate and most multivariate methods, often requiring more sophisticated approaches [e.g., 49].

The major limitation of this investigation concerns missing monthly records, which are interpolated with monthly median values from pertinent CSCOM service areas as described in *Methods*. A missing monthly record comprises the complete lack of figures for all diseases and age categories within a single CSCOM service area and month—i.e., a missing monthly record from a single CSCOM service area does not imply the simultaneous lack of records for the other 16 CSCOM service areas. Consequently, results presented herein carry admonitions concerning: *i*) the potential bias of calculated annual consultation rates and dispersion narrowing (Table 2); as well as, *ii*) the introduction of TS periodicity.

The potential bias of calculated annual consultation rates diminish with adoption of median and IQR values to robustly measure centrality and dispersion, respectively. In the TS context, records from 3 (from a total of 17) CSCOM service areas are excluded from analyses (*Methods*). Disease- and age category-specific amalgamated TS comprise 14 data sets, each from an eligible CSCOM service area; hence, a composite monthly TS observation reflects an aggregate of 14 CSCOM service area entries. Missing records distribute approximately normally across CSCOM service areas (Shapiro-Wilk normality test: *W* = 0.9193; *p-value* = 0.1436) and approximately uniformly through the investigational period (*Methods*). The percentage of missing monthly records in the amalgamated TS is 13.6 %, generally less than 2% *per* year (*Methods*). The only exception manifests in the, practically reconstructed, year of 1997 that was employed for program initialization—nonetheless, this is minimally consequential because program initialization, otherwise, would reflect the customary (and arbitrary) ‘opinion of an expert’. Consequently, artificial TS periodicity introduction subsequent to the interpolation procedure is highly improbable. Further details on data pre-processing appear in the *Methods* section.

### Conclusion

Notwithstanding the paucity of published MHW-based infectious disease TS forecasting investigations, these results suggest that the exceptional forecasting performance of the MHW method in econometrics and beyond since the 1950s may be capitalized by the public health sector. The MHW method is operationally simple, general, reasonably accurate, and available in many commercially and freely available software and programming environments. It produces forecasts for very distinct diseases (diarrhea, acute respiratory infection, and malaria) without disease-specific tailoring of forecasting method, adapting *on-line* to underlying disease TS perturbations. Finally, this method does not require covariates to forecast diarrhea, acute respiratory infection, and malaria TS under fully- or partially-stable transmission. Therefore, the MHW method is a strong general-purpose forecasting method candidate for the aforementioned non-stationary epidemiological TS. These forecasts could improve infectious diseases management in the district of Niono, Mali, and elsewhere in the Sahel.

## Methods

### Study setting

This longitudinal retrospective (01/1996–06/2004) TS investigation is conducted in the district of Niono, Mali (Fig. 1). Panel A in Fig. 1 is a satellite image that portrays Mali, with a projected population of 12 million in 2004 [40], along with its neighboring West-African countries. Panel B corresponds approximately to the enlarged red demarcation seen in panel A. The district of Niono (330 km northwest of Bamako, 100 km north of the Niger River, in the Segou region) is depicted within the red rectangle in panel B. The district of Niono is a model location to test disease TS forecast and early warning system feasibility because it shares epidemiological similarities with other regions in the Sahel where poverty- and disease-induced morbidity and mortality are rampant. Additionally, available monthly consultation records for 20 diseases (including diarrhea, ARI, and malaria) are monthly resolved in the district of Niono.

### Data pre-processing

The review of monthly clinical records is part of a larger study on climate and health (“Putting climate in the service of public health”) that is approved by the “Columbia University Medical Center Institutional Review Board” (N.Y., U.S.A.) and the “Ethics Committee of the Mali National Medical School ” (Bamako, Mali). Patient privacy is secluded from inadvertent (or deliberate) violations because consultation records comprise monthly summaries that lack information with which individual patients may be identified. The assembled monthly data set (01/1996–06/2004) contains consultation records for 20 diseases, which are tabulated by gender and age category, from 17 CSCOM service areas [39] within the district of Niono.

However, only data for diarrhea, ARI, and malaria are herein analyzed beyond frequency description because these diseases inflict most morbidity and mortality in this district. The national estimates for age composition are: 4.0% [0–11 months], 15.3% [1–4 years], 28.1% [5–15 years], and 52.6% [>15 years] [40]. The total projected population (2004) for the district of Niono is 278 741 individuals [39], [40]. Consultation records from each CSCOM service area are adjusted with the annual national population growth rate of 3.2% [40] before missing records (**Tables 4** & **5**) are interpolated by CSCOM-specific monthly median values.

**Table 4:** Demographic and consultation record descriptions.

CSCOM	Population (2004)	Time-series period	Missing dates	Missing months	% missing
**Boh**	7105	01/1996–06/2004	-	0	0.00 (0.00)
**Bolibana**	18321	01/1996–06/2004	1997	12	0.76 (0.84)
**Cocody**	6021	01/1996–06/2004	-	0	0.00 (0.00)
**Debougou**	25603	01/1996–06/2004	1997, 1998 (3)	15	0.94 (1.05)
**Diabaly**	16974	01/1996–06/2004	1997	12	0.76 (0.84)
**Diakiwere**	12269	01/1996–06/2004	1999 (3), 2003 (3)	6	0.38 (0.42)
**Dogofry**	24172	01/1996–06/2004	1997, 1998 (1)	13	0.82 (0.91)
**Fassoun**	5837	01/1996–12/1999	1997, 1999 (9)	21	1.33 -
**Kourouma**	8186	01/1996–06/2004	1997, 2001	24	1.52 (1.68)
**Molodo**	18379	01/1996–06/2004	1997, 2003 (6)	18	1.14 (1.26)
**Nampala**	7972	01/1996–06/2004	1996 (4), 1997, 1999 (9)	25	1.58 (1.75)
**Nara**	24161	01/2000–06/2004	2000, 2001, 2002, 2003 (6)	42	2.65-
**Pogo**	11893	01/1996–06/2004	1997, 2003 (3)	15	0.94 (1.05)
**Siribala**	22745	01/1996–06/2004	1997, 2001 (3)	15	0.94 (1.05)
**Sokolo**	14672	01/1996–06/2004	1997, 1999 (3)	15	0.94 (1.05)
**Werekela**	14431	01/1996–06/2004	1996, 1997	24	1.52 (1.68)
**Niono**	40000	01/2000–06/2004	2000, 2001 (2), 2002 (1)	16	1.01 -
**Total**	278741 (208743)	1584 (1428) months	-	272 (194)	17.2 (13.6)

The projected number of individuals (2004) served by each community health center (CSCOM) service area within the district of Niono, Mali, is tabulated under the *Population* heading. Potential records are listed under *Time-series period*. Unavailable CSCOM service area records appear under *Missing dates*—the number of missing monthly records for each year is listed in parenthesis otherwise records for the whole year are missing. These are totaled under *Missing months* and expressed as percentages from the total number of possible monthly records (across all CSCOM service areas) under the *% missing* heading. Monthly consultation records from each CSCOM service area are adjusted with the annual national population growth rate before missing records are interpolated by CSCOM-specific monthly median values. The total projected population (2004) for the district of Niono is 278 741 individuals, growing 3.2% annually according to regional [39] and national [40] estimates. In the context of consultation rates, years with more than 6 missing monthly records are excluded from median and inter-quartile-range annual consultation rate calculations. Additionally, inter-quartile-range calculations require records from at least 3 eligible years. In the time-series context, however, the projected district population (2004) reduces from 278 741 to 208 743 individuals upon record exclusion from Fassoum, Nara, and Niono CSCOM service areas—which do not span the entire investigational period and possess excessive missing monthly records. Values that are reported in parenthesis under the headings of *Population* (2004), *time-series period*, *Missing months*, and *% missing* reflect record exclusion from Fassoum, Nara, and Niono CSCOM service areas. After record exclusion, the remaining 14 CSCOM service areas contribute 1428 possible monthly records to the amalgamated time-series, i.e., 14 records *per* month. Of note, the Niono CSCOM service area, which includes the district center and immediate periphery, is one of the 17 CSCOM service areas within the district of Niono, Mali.

**Table 5:** Monthly missing record distribution.

Frequency (%)										
Year	1996	1997	1998	1999	2000	2001	2002	2003	2004	total
% missing	1.0 (1.1)	9.1 (9.2)	0.3 (0.3)	1.5 (1.1)	1.5 (0.0)	1.8 (1.1)	0.8 (0.0)	1.1 (0.8)	0 (0.0)	17.2 (13.6)

A total of 272 monthly entries are missing from 1584 possible records, accounting for 17.2% of missing data across all community health center (CSCOM) service areas in the district of Niono, Mali, during the investigational period (01/1996–06/2004). Percentage of missing monthly records after the exclusion of Fassoum, Nara, and Niono CSCOM service areas are listed in parenthesis. The annual percentage of missing monthly records is generally less than 2% *per* year. The only exception manifests in the practically reconstructed year of 1997 that was employed for program initialization. Consequently, artificial time-series periodicity introduction subsequent to the interpolation procedure is highly improbable.

After adjustment for population growth and missing value interpolation, age category-specific median annual diarrhea, ARI, and malaria consultation rates (plus their IQR values) are calculated for each CSCOM service area to assess age effects on consultation distributions. These consultation rates are calculated, and expressed, as the number of newly diagnosed cases *per* 1000 individuals in an age category during a year. In the TS context, 3 CSCOM service areas are excluded from the TS analyses because of limited record availability, i.e., these CSCOM TS do not span the entire investigational period, contain excessive missing monthly records and hence, do not meet the inclusion criterion (Table 4): Niono and Nara CSCOM service areas (01/2000–06/2004) as well as Fassoum CSCOM service area (01/1996–12/1999). Of note, the Niono CSCOM service area, which includes the district center and immediate periphery, is one of the 17 CSCOM service areas within the district of Niono. Upon record exclusion, the considered district population reduces to 208 743 individuals (Table 4). Sequentially, the adjusted monthly consultation records for the remaining 14 (from a total of 17) CSCOM service areas are amalgamated according to age category and disease (diarrhea, ARI, and malaria), converted to rates, and then submitted to TS analyses. TS consultation rates are expressed as the number of newly diagnosed cases *per* 1000 individuals in an age category during a month.

### Time-series forecasts

The resulting TS are fitted with the MHW method. The MWH method is a versatile non-parametric technique (not to be confused with a statistical model, which has a defined error structure) that requires neither stationarity nor ‘strict’ linearity to produce contemporaneous *on-line* TS forecasts at variable horizons, *h* [37], [38]. The MHW method adapts to underlying alterations in disease dynamics and revises its estimates *on-line*, i.e., with the accumulation of new observations. This adaptability is essential for epidemiological forecasting that depends on historical data because interventions (e.g., prophylaxis and medical treatment) almost ubiquitously plague longitudinal retrospective TS. Last but not least, exponential smoothing methods (e.g., MHW) generally perform better than statistically more sophisticated alternatives according to a recent forecasting competition, which compared several forecasting techniques across more than a thousand TS [50].

The MHW optimizes *via* minimization of the squared prediction error for the 1-month horizon forecast (*h* = 1) with a modified Quasi-Newton non-linear optimization method referred to as L-BFGS-B, i.e., the limited memory (L) Broyden-Fletcher-Goldfarb-Shanno (BFGS) method for bounded (B) variables [51], [52]. In Equation 1, *F*(*y_t_*_+*h*_|*I_t_*) is the *h*-month horizon forecast for the observed TS {*y_t_*} conditioned on the information set (*I_t_*) that accumulates until time *t*; *l_t_* and *r_t_* are the TS level and trend components, respectively, as previously defined; and, *s_m|t–p+h_* is the unitless, monthly seasonal factor that is estimated at time *t–p+h* (for *h*≤12). The index *m* = [1], [12] reflects the season of the forecasting horizon *h* whereas the period *p* is herein defaulted to 12 months. Possible lower-frequency harmonics, i.e., inter-annual fluctuations, are handled as level and trend components by the MHW method because the limited temporal window considered (01/1996–06/2004) in this investigation precludes stable estimation of oscillations with periods much longer than 12 months.
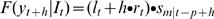
(1)


The MHW method revises estimates recursively as new observations accumulate *ad infinitum* with Equations 2, 3, and 4 where *α*, *β*, and *γ* are the smoothing pseudo-parameters for *l_t_*, *r_t_*, and *s_m_*_|*t*_, respectively, as defined in the *Results* section. The MHW smoothing pseudo-parameters are embedded in recursive equations (2, 3, and 4) whose roles resemble exponential kernel smoothing of *l_t_*, *r_t_*, and *s_m_*_|*t*_ [53].
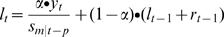
(2)

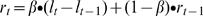
(3)

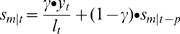
(4)


In the simplest case, i.e., in the absence of trend (*β* = 0) and periodicity (*γ* = 0), the MHW method reduces to a simple exponential smoothing filter that has been described in terms of the Nadaraya-Watson (one-sided) exponential kernel [53]. Other special cases arise if *γ* = 0 or *β* = 0, namely when this exponential method lacks a seasonal or a trend component, respectively. Large MHW pseudo-parameters confer greater weights to recent information and effectively shorten the smoothing ‘memory’, i.e., the recent-past has a more pronounced influence on estimated *l_t_*, *r_t_*, and *s_m_*_|*t*_ values than does the distant-past. Fitting is initialized by setting *α*, *β*, and *γ* to 0.3 since their initial choice is trivial—typical and possible pseudo-parameters are 0<*α*, *β*, and *γ*<0.5 and 0<*α*, *β*, and *γ*<1, respectively. Initial values for *l_t_*, *r_t_*, and *s_m_*_|*t*_ obtain automatically from a simple moving-average decomposition that employs a minimum of 3 initial TS periods (i.e., 36 months *per* default), the feasible specification of additional periods (e.g., *p* = 4, 5…) notwithstanding [37], [38], [52], [54]. Forecasts are produced only after 3 TS periods—subsequent to initialization with the first period *p* (i.e., the first 12 months) and fitting of the remaining 24 months.

### Residual time-series bootstrapping

Not only does the MHW method produce accurate forecasts but it also naturally decomposes {y*_t_*} into {*l_t_*}, {*r_t_*}, {*s_m_*_|*t*_}, and {*z_t_*}, which are shorter than the observed TS by exactly *p* observations. However, the lack of an underlying statistical model hinders the estimation of 95% PI values. Thus, median forecasts and their empirical 95% PI values are estimated from probability density functions that are generated at each time *t* for horizon *h* by uniform bootstrapping (*B* = 500) the approximately serially uncorrelated {*z_t_*} TS distribution [55]. The {*z_t_*} TS distribution, which elongates as new observations and their forecasts accumulate, is calculated with Equation 5;
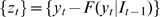
(5)where
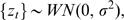
i.e., {*z_t_*} is an approximately stationary white-noise process with mean 0 and variance *σ* ^2^. The first *p* original TS observations (for which fitted values are unavailable) are concatenated with shorter pseudo-TS of length *t–p*, which are generated *via B* = 500 {*z_t_*} TS bootstraps and subsequent TS reconstruction with the inverse function of Equation 5. This ensures that observed TS and reconstructed pseudo-TS have the same length. The absolute value of the rare negative monthly pseudo-TS value is enforced because this forecasting method handles non-negative values only. Each reconstructed pseudo-TS of length *t* is fitted with the MHW method; forecast probability density functions are produced for *h* = 2 and *h* = 3, from which median forecasts and their empirical 95% PI values yield. Quantiles are calculated with *p*(*k*) = (*k*−1)/(*n*−1) *via* linear interpolation between the *k^th^* order statistics and *p*(*k*) where *n* is the sample size; further information is available in “R: language and programming environment” [52] and references therein. Reported values for *α*, *β*, and *γ* reflect the median and IQR values of their probability density function upon fitting *B* = 500 full-length pseudo-TS.

### Forecasting accuracy and precision

Forecasting accuracy is calculated as MAPE values between observed monthly consultation rates and their median forecasts. The MHW method performance is compared against that of the also operationally simple SA_3_ forecasting benchmark, which is recommended in Abeku *et al.* [32]. SA_3_ forecasts are generated by correcting seasonal averages with the mean deviation between the three most recent seasonal forecasts and their observed TS values [32] hence, capturing recent TS trend vis-à-vis reducing statistical variation. Finally, precision is identified with 95% PI values. Large MAPE (or PI) values imply low accuracy (or precision) and *vice-versa*.
